# Fast 3D dosimetric verifications based on an electronic portal imaging device using a GPU calculation engine

**DOI:** 10.1186/s13014-015-0387-7

**Published:** 2015-04-11

**Authors:** Jinhan Zhu, Lixin Chen, Along Chen, Guangwen Luo, Xiaowu Deng, Xiaowei Liu

**Affiliations:** School of Physics and Engineering, Sun Yat-sen University, 510275 Guangzhou, China; Sun Yat-sen University Cancer Center; State Key Laboratory of Oncology in South China; Collaborative Innovation Center for Cancer Medicine, 510060 Guangzhou, China

**Keywords:** 3D dosimetric verification, EPID, GPU

## Abstract

**Purpose:**

To use a graphic processing unit (GPU) calculation engine to implement a fast 3D pre-treatment dosimetric verification procedure based on an electronic portal imaging device (EPID).

**Methods:**

The GPU algorithm includes the deconvolution and convolution method for the fluence-map calculations, the collapsed-cone convolution/superposition (CCCS) algorithm for the 3D dose calculations and the 3D gamma evaluation calculations. The results of the GPU-based CCCS algorithm were compared to those of Monte Carlo simulations. The planned and EPID-based reconstructed dose distributions in overridden-to-water phantoms and the original patients were compared for 6 MV and 10 MV photon beams in intensity-modulated radiation therapy (IMRT) treatment plans based on dose differences and gamma analysis.

**Results:**

The total single-field dose computation time was less than 8 s, and the gamma evaluation for a 0.1-cm grid resolution was completed in approximately 1 s. The results of the GPU-based CCCS algorithm exhibited good agreement with those of the Monte Carlo simulations. The gamma analysis indicated good agreement between the planned and reconstructed dose distributions for the treatment plans. For the target volume, the differences in the mean dose were less than 1.8%, and the differences in the maximum dose were less than 2.5%. For the critical organs, minor differences were observed between the reconstructed and planned doses.

**Conclusions:**

The GPU calculation engine was used to boost the speed of 3D dose and gamma evaluation calculations, thus offering the possibility of true real-time 3D dosimetric verification.

## Background

As the frequency of dose prescriptions and the complexity of radiotherapy techniques increase, so too do the demands for accurate and efficient methods of verifying the doses delivered to patients. Hence, dosimetric verification is a prerequisite to ensure correct treatment planning and delivery. In recent years, an increasing number of reports have begun to focus on the 3D dosimetric verification of intensity-modulated radiation therapy (IMRT) treatment plans [1-3]. Most planar dosimetric verifications are based on uniform phantoms, but this method cannot be used to determine the algorithm error of a treatment planning system (TPS) with respect to inhomogeneities. Nelms *et al.* have reported that there is a lack of correlation between conventional IMRT quality assurance (QA) performance metrics (gamma pass rate) and dose errors in anatomical regions of interest. There is no clear relation between the gamma pass rate and the error in dose–volume histograms (DVHs) [4]. 3D dosimetric verifications can yield more intuitive anatomy-based 3D dose differences for clinical verifications and other detailed evaluation indices, such as the DVH.

Because of their sub-millimeter resolution, real-time response and lower workload, electronic portal imaging devices (EPIDs) are often utilized for pre-treatment and in vivo measurements [1,2,5-7]. There are two approaches to using an EPID for 3D dosimetric verifications. One technique is to calculate the 3D dose distributions based on the EPID measurement using a TPS. Steciw *et al.* have reported the use of a TPS to calculate dose distributions based on the fluences of treatment fields that were measured using an EPID [8]. This method can be easily performed in clinical practice because no effort is required for the independent dose-calculation algorithm and model commissioning. However, the effect of the TPS calculation errors cannot be estimated. Moreover, not all commercial TPSs have functions to export fluence maps or to calculate doses based on input fluence maps. The other technique is to use an independent dose-calculation algorithm. Wendling *et al.* have reported the use of the “back-projection” algorithm for this purpose [9]. This method is simple and has been used clinically in their institution [6]. The model is “water-equivalent,” as the parameters are determined under homogeneous conditions. For verifications of lung treatments in which inhomogeneities are important, a correction method known as the “in aqua vivo” method is applied [10]. Elmpt *et al.* have implemented 3D dose verifications based on Monte Carlo simulations. If implemented correctly, such simulations can provide, in principle, the highest accuracy that is currently available [2]. Recent publications have reported the implementation of a pencil-beam algorithm for fast 3D dosimetric verifications [11-13]. Because of the simplicity of this pencil-beam algorithm, its computation speed is very fast. However, its limitations cause it to be unsuitable for application to inhomogeneous tissues [14].

In clinical applications, whether for pre-treatment or for in vivo dosimetric verifications, two aspects must be considered: first, the computation time must be considered, with the potential goal of real-time verification, and second, the accuracy in inhomogeneous tissues must be examined. The collapsed-cone convolution/superposition (CCCS) [15] algorithm is often used in radiotherapy TPSs. The calculation accuracy of the CCCS algorithm for inhomogeneous tissues is acceptable. A recent report demonstrated that the results produced by the CCCS dose engine that is used in the TomoTherapy® TPS are consistent with Monte Carlo results within 1%/1 mm in water and 2%/2 mm in the lung, except in cases involving a small field (1.25 cm × 1.25 cm) and a tissue–air interface [16]. Thus, the use of the CCCS algorithm is a compromise between the computation time and the accuracy in inhomogeneous tissues.

Although the CCCS algorithm incorporates the collapsed-cone approximation, which allows the calculation complexity to be reduced in comparison with full-volume point-to-point convolution, it is still computationally demanding, especially for IMRT, which involves many fields or segments. Graphic processing units (GPUs) are increasingly being used for scientific applications, including 3D dosimetric calculations, that can be accelerated by exploiting the parallel architecture and unprecedented computing power density of GPUs [17-20]. Quan *et al.* have reported that a GPU algorithm using exponential cumulative–cumulative kernels (CCKs) is 1000–3000 times faster than a highly optimized single-threaded CPU implementation using a tabulated CCK [19]. Computation times on the order of seconds can be achieved. Thus, a GPU-based CCCS algorithm demonstrates good prospects for achieving true real-time 3D dosimetric verifications.

An EPID can be used to determine the delivery errors of treatment plans, and an accurate independent dose-calculation algorithm can be used to estimate the errors of TPS dose calculations, including scenarios involving the treatment of inhomogeneous sites such as lung tissues and oral-nasal cavities, and to cross-check the TPS modeling, which is strongly dependent on the input measurement data. The computation speed can be boosted via GPU implementation. The GPU implementation of fast EPID-based 3D pre-treatment dosimetric verifications is presented in this paper. The GPU algorithm includes the deconvolution and convolution method for the fluence-map calculations, the CCCS algorithm for the 3D dose calculations and the 3D gamma evaluations. The combination of an EPID-based approach and a GPU-based approach can effectively reduce the measurement workload and calculation time that are required for pre-treatment verifications. Furthermore, real-time IMRT dosimetric verifications and dose-guided radiation therapy pose stringent requirements in terms of measurement and calculation-time capabilities. The combination of features provided by EPIDs and GPUs can satisfy these requirements.

## Methods

### EPID image acquisition

A Trilogy linear accelerator system (Varian Medical Systems, Palo Alto, CA, USA) with an aS1000 EPID (Varian Medical Systems, Palo Alto, CA, USA) was employed in this study. Both 6 MV and 10 MV photon beams were used for all measurements. The physical size of the EPID was as follows: the EPID had a sensitive area of 40 cm × 30 cm in size, and the effective pixel size was 0.04 cm × 0.04 cm. The pre-treatment EPID measurement was performed in air without a phantom/patient. Because the EPID images were used as the input for subsequent dose calculations, it was necessary to completely capture the treatment fields in the cross-plane direction. The effective source-to-detector distance was set to 140 cm. Image acquisition was performed in integrated mode, and offset correction, gain correction and pixel correction were performed for each image. Because of the robotic arm that is located directly beneath the sensitive area of the Varian EPID, the backscatter from the arm can have a deleterious effect when the EPID is used for dosimetric purposes [21-23]. The backscatter correction kernel was modeled as a radially symmetric Gaussian and applied to the raw EPID data [24]. To ensure positioning accuracy during image acquisition, a gantry-angle-dependent correction associated with the panel position displacement was applied [25]. The EPID displacement was measured by comparing the image acquired at each gantry angle to the 0° image in the 10 cm × 10 cm field.

### Fluence-map calculation

The pixel values of the EPID images, which captured the treatment fields during air delivery, were reconstructed into fluence maps at a source–axis distance (SAD) of 100 cm using the deconvolution and convolution method.

In our previous studies [26], the EPID, which consists of several layers, was treated as a uniform water-equivalent phantom. The relationship between the pixel values of the EPID images and the dose values on the central plane of the 20-cm-thick virtual water phantom, which was placed at a source-to-surface distance (SSD) of 90 cm, can be represented as follows:

1 $$ {D}_{ij}={c}_{\mathrm{ad}}\cdot {P}_{ij}{\otimes}^{-1}{K}_{ij}^{\mathrm{EPID}}\otimes {K}_{ij}^{\mathrm{Water}} $$

where ⊗ and ⊗ ^− 1^ denote the convolution and deconvolution operators, respectively. Every pixel in the EPID image is referred to by its indices *i* and *j*; *P*_*ij*_ is the pixel value of the EPID image that is projected to the phantom level, *D*_*ij*_ is the dose at the phantom level, *c*_ad_ is the absolute dose calibration factor, ${K}_{ij}^{\mathrm{EPID}}$ is the EPID scatter kernel that is used to obtain the incident fluence map, and ${K}_{ij}^{\mathrm{Water}}$ is the convolution kernel that is used to calculate the dose distribution in the water phantom.

In this study, this model was used to reconstruct the EPID image into a fluence map; thus, equation (1) was simplified as follows:

2 $$ {D}_{ij}={c}_{\mathrm{ad}}\cdot {P}_{ij}{\otimes}^{-1}{K}_{ij}^{\mathrm{EPID}} $$

with

3 $$ {K}_{ij}^{\mathrm{EPID}}=\left(1-c\right) \exp \left(\hbox{-} {\mu}_{\mathrm{S}}{d}_{ij}\right)+c\cdot \exp \left(\hbox{-} {\mu}_{\mathrm{L}}{d}_{ij}\right) $$

Here, *d*_*ij*_ is the distance from the center of the kernel to a pixel *ij. μ*_S_ and *μ*_L_ are the attenuation coefficients, which depend on the energy and the material. There are two terms in this equation. The first term, which contains *μ*_S_, is the short-range primary dose component. It describes the energy per unit mass imparted by the charged particles released as a result of the first interaction of a primary photon. The second term, which contains *μ*_L_, is the long-range scatter dose component. It describes the energy imparted by charged particles set in motion by secondary photons, i.e., by scattered, bremsstrahlung and annihilation photons.

Because of the broad distribution of photon energies within the phantom and the EPID, there will be an effective attenuation coefficient for the energy spectrum. Instead of the use of multiple monoenergetic kernels based on the beam’s energy spectrum, *μ*_S_ and *μ*_L_ were treated as free parameters in the fit procedure. For each nominal energy of the linear accelerator, the parameters of ${K}_{ij}^{\mathrm{EPID}}$ are different. These parameters were determined using the central point dos × es, which were measured using an ion chamber in air for fields of 3 cm × 3 cm to 20 cm × 20 cm. The parameters of the ${K}_{ij}^{\mathrm{EPID}}$ kernel that yielded the minimum variance could thus be determined via the golden-section search algorithm [27]. Because the structure of an EPID is invariant, these parameters were constants once the fit procedure had been completed.

A simple exponential kernel was introduced to reduce the noise arising from the application of the deconvolution operator, and a Gaussian function was used to correct the “horns” from the beam profile.

To improve the calculation speed, a fast Fourier transform (FFT) was used. The FFT was applied using the CUFFT library from the NVIDIA® CUDA™ library, which is highly optimized for computing parallel FFTs using an NVIDIA GPU.

The EPID-based fluence maps for fields of 3 cm × 3 cm to 20 cm × 20 cm were compared to the ion-chamber results. The S_c_ factor and the dose profiles in air were obtained using a 0.13-cc ion chamber (IBA Dosimetry GmbH, Schwarzenbruck, Germany) with a buildup cap by scanning an empty 3D water tank (Blue Phantom, IBA Dosimetry GmbH, Schwarzenbruck, Germany) at an SAD of 100 cm.

### 3D dose reconstruction using a GPU

#### 3D dose-calculation algorithm

The fluence-map-based CCCS algorithm includes two independent components: the calculation of the total energy released per mass (TERMA) and the convolution/superposition (CS) calculation of the energy deposition [15].

The TERMA calculation models the primary photons that interact with the material. Because the CCCS algorithm was used for EPID-based 3D dosimetric verifications in this study, a measured source model was used for the TERMA calculation instead of a theoretically calculated source model, which must account for all elements of the system, including the flattening filter, the collimator system, the multi-leaf system, etc.[12]. The EPID-based fluence maps, which included the intensity of the beam emitted from the head of the linear accelerator, were taken as the input. The spectrum of the beam was obtained from the phase-space files that were simulated for the Varian Trilogy 6-MV and 10-MV photon beams using BEAMnrc [28], and the geometry and material parameters of the linear accelerator were obtained from the manufacturer. The TERMA calculation was performed based on the electron density and the linear attenuation coefficient of water during the ray tracing, the latter of which was obtained from the National Institute of Standards and Technology (NIST). The calculation had a complexity of O (N^3^) for N^3^ voxels. Each voxel was calculated by one GPU thread.

Three types of kernel expressions can be used in the discretization of the CCCS algorithm: the differential kernel (DK), the cumulative kernel (CK) and the cumulative–cumulative kernel (CCK) [29]. Lu *et al.* reported that the CCK is the most accurate because of its inherent voxel integration; therefore, the CCK was used in this study. The CCK was pre-calculated from discrete Monte Carlo kernels, which were simulated using the edknrc program [30] for uniform water. The lookup method based on tabulated kernels was used in the calculation. The kernels were scaled in accordance with the electron densities between the interaction point and the receiving points to allow the water kernels to be applied to arbitrary inhomogeneous media [15,29]. Considering that the kernel intensity had an angular distribution, the collapsed-cone directions were divided into non-uniformly sampled zenith angles to improve the calculation speed. The collapsed-cone directions were divided as follows: 22 uniformly sampled angles from 0° to 44°, 1 angle from 44° to 50°, 9 uniformly sampled angles from 50° to 95°, 1 angle from 95° to 120° and 3 uniformly sampled angles from 120° to 180°. The calculation also included 8 uniformly sampled azimuthal angles. In total, 280 collapsed-cone directions were used. Each voxel was calculated in one kernel direction by one GPU thread and then looped through all directions. This method is well suited to GPU implementation because adjacent threads can be used to access adjacent memory locations [19].

#### Algorithm heterogeneity evaluation

**Figure 1:**
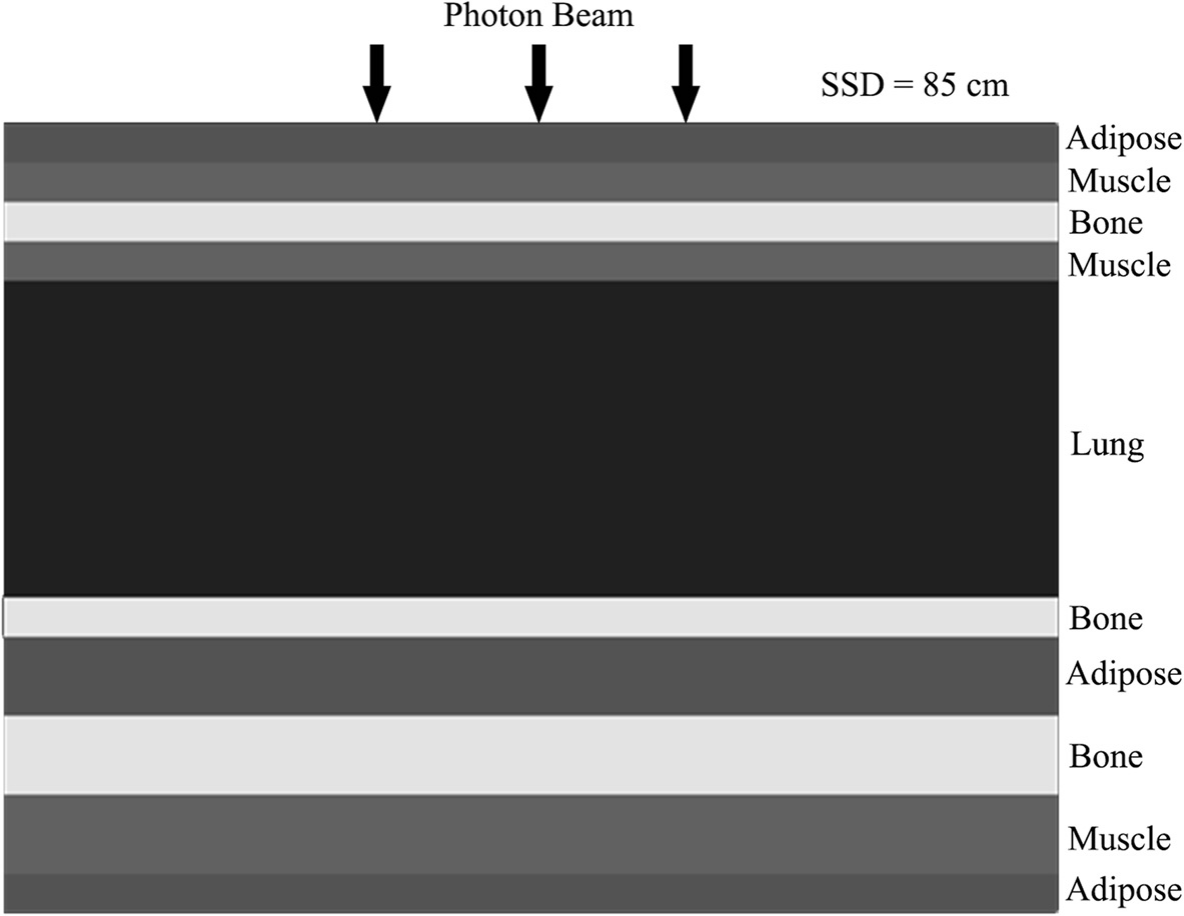
A schematic diagram of the heterogeneous slab phantom and setup.

A slab phantom similar to that used by Ahnesjo [15] was employed in this study. This phantom consisted of slabs of simulated tissues (adipose (A, density = 0.920 g cm^−3^), muscle (M, density = 1.040 g cm^−3^), bone (B, density = 1.850 g cm^−3^) and lung (L, density = 0.250 g cm^−3^)). The thickness of the phantom was 30 cm, and its structure is illustrated in Figure 1. The source-to-surface distance (SSD) was 85 cm. The sizes of the evaluation fields were 3 cm × 3 cm, 10 cm × 10 cm and 20 cm × 20 cm. The calculation methods included (1) the GPU-based CCCS algorithm, (2) a full Monte Carlo simulation and (3) the analytical anisotropic algorithm (AAA). The size of the calculation grid for all methods was 3 mm × 3 mm × 3 mm. The EGSnrc user code DOSRZnrc [31], with the phase-space files mentioned in the previous section, was used to simulate the dose delivered to the material. Because the treatment plans were created and optimized using Eclipse v10.0.28 (Varian Medical Systems, Palo Alto, CA, USA), which uses the AAA for dose calculations, the AAA was also considered in the evaluation.

### 3D dosimetric verifications for example clinical treatment plans

#### Treatment planning

One head-and-neck IMRT treatment plan and one lung IMRT treatment plan were chosen for 6 MV dosimetric verification. One lung IMRT treatment plan and one prostate IMRT treatment plan were chosen for 10 MV dosimetric verification. The treatment plans were created and optimized using Eclipse v10.0.28, and the AAA was used as the dose-calculation algorithm. The size of the calculation grid was 3 mm × 3 mm × 3 mm. Nine radiation fields were used for the head-and-neck and prostate treatment plans, and five radiation fields were used for the lung treatment plan. The optimization objective functions of the targets were generally set such that the planning target volume (PTV) received at least 95% of the prescribed dose. The optimization objective functions of the other normal tissues were set following clinical requirements. The prescribed doses for the 6 MV head-and-neck, 6 MV lung, 10 MV lung and 10 MV prostate plans were 68.2 Gy, 66.0 Gy, 66.0 Gy and 67.5 Gy, respectively.

#### Dosimetric verifications

The 3D dose-reconstruction results that were obtained based on the EPID were compared to the TPS results. Because a measured source model was used in our dose calculation, it was difficult to directly quantify the differences in the dose-calculation algorithms and the sources of delivery error for these two different methods. It is well known that dose-calculation algorithms, including the CCCS algorithm and the AAA, are accurate in a uniform water phantom [15,29,32]. Therefore, the material within the patient’s body structure was first overridden to water (mass density 1.0 g cm^-3^). This aided in excluding any inherent difference between the dose-calculation algorithms when quantifying the effect of the delivery error. The TPS was used to recalculate the dose distributions in the overridden water phantom for the verification plans, and the EPID-based fluence maps were used to reconstruct the dose distributions in the same phantom. The comparisons were performed with respect to the following: 1) Dose differences, including the differences in the mean doses (D_mean_) and the maximum doses (D_max_) to the targets and the organs at risk (OARs). The percent dose differences were calculated using the following equations:

4 $$ Dif{f}_{\mathrm{mean}}=\left({D}_{\mathrm{mean}}^{\mathrm{EPID}}-{D}_{\mathrm{mean}}^{\mathrm{TPS}}\right)/{D}_{\mathrm{prescribed}}\times 100\% $$

5 $$ Dif{f}_{\max }=\left({D}_{\max}^{\mathrm{EPID}}-{D}_{\max}^{\mathrm{TPS}}\right)/{D}_{\mathrm{prescribed}}\times 100\% $$

2) 3D gamma evaluations. The same areas that were used for the dose comparisons were treated as the regions of interest (ROIs). The dose grid size was resampled to 1 mm × 1 mm × 1 mm before the gamma evaluation was performed. We set 3% of the maximum dose as the dose criterion and 3 mm as the distance criterion (henceforth referred to as the 3%/3 mm criterion), and a tighter 2%/2 mm criterion was also evaluated. A statistical evaluation was performed to determine the point at which the dose was greater than 10% of the maximum dose.

The 3D dose-reconstruction results and the TPS results from the original patient CTs were also considered for comparison. These comparisons were performed with respect to the following: 1) Dose differences, including those in the D_mean_ and D_max_ values of the targets and OARs, and 2) 3D gamma evaluations. The evaluation criteria were the same as those listed above.

One disadvantage of the 3D gamma evaluation is its long computation time [33,34]. With the dose grid size resampled to 1 mm × 1 mm × 1 mm, the computation time using a fast algorithm for the body gamma evaluation would be approximately 1 minute [34]. However, applying a GPU implementation of the gamma evaluation can reduce the computation time [35]. Because GPU texture memory provides high-efficiency trilinear interpolation for 3D data, the dose data were bound to the GPU texture memory for the resampling process. The gamma index of each voxel was calculated by a separate GPU thread. Voxels with dose values lower than the threshold were excluded from the calculation process.

All calculation algorithms discussed above were implemented in Visual C++. All computations were performed on a notebook with an Intel Core i7-4700MQ 2.40 GHz CPU and an NVIDIA GTX-765 M video card.

## Results

### Evaluations of EPID-based fluence maps

**Figure 2:**
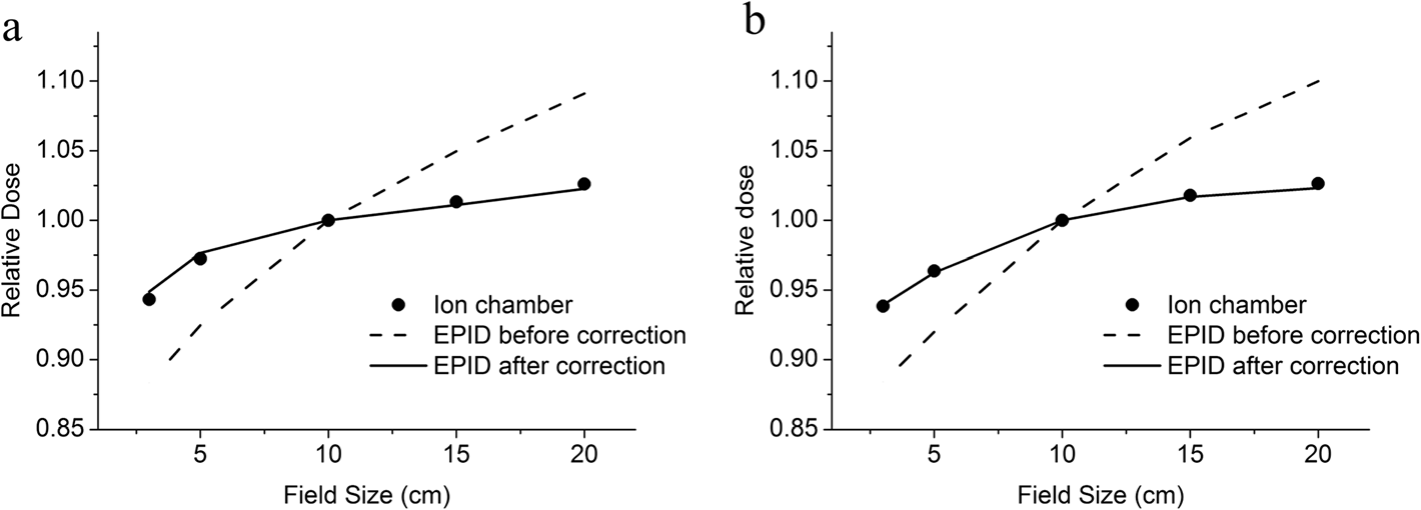
The central point dose values of the square-field results for the ion chamber (black points), the EPID before correction (dashed lines) and the EPID after correction (solid lines) for **a**) the 6 MV photon beam and **b**) the 10 MV photon beam. The relative doses were normalized to the central dose of the 10 cm × 10 cm field.

**Figure 3:**
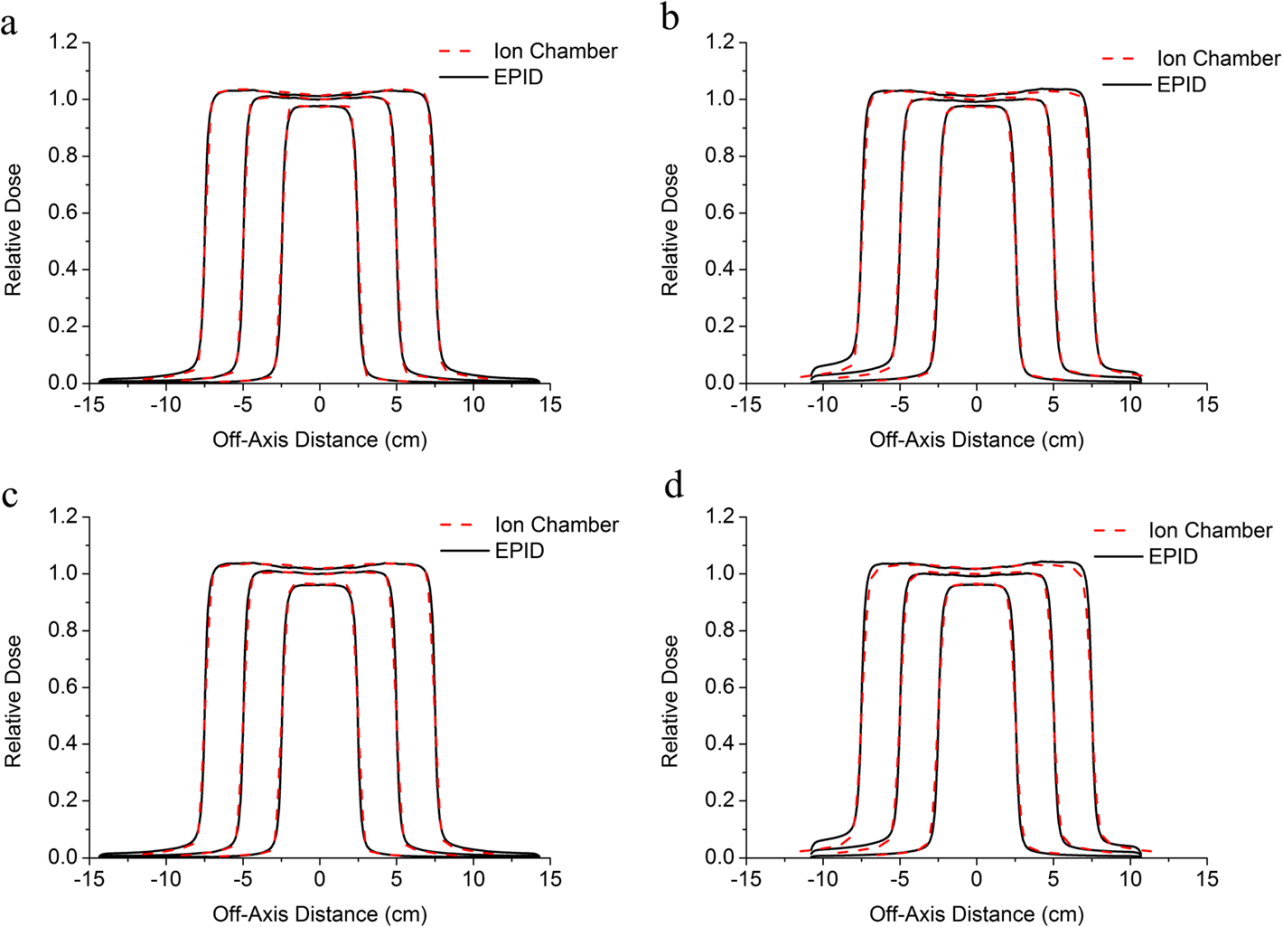
The fluence profiles on the central axis determined using the EPID (black solid lines) and using the ion chamber (red dashed lines): **a**) the cross-plane central axis for the 6 MV beam; **b**) the in-plane central axis for the 6 MV beam; **c**) the cross-plane central axis for the 10-MV beam; and **d**) the in-plane central axis for the 10 MV beam. The relative doses were normalized to the central dose of the 10 cm × 10 cm field.

Figure 2 shows the central-point relative dose values in air that were measured using the ion chamber and the EPID for square fields of various sizes. When our deconvolution model correction was applied to the raw EPID data, the maximum difference between the EPID results and the ion-chamber measurements was less than 0.5% for both the 6 MV and 10-MV photon beams. Figure 3 shows the fluence profiles on the central axis that were measured using the ion chamber and the EPID after correction. The agreement was good for both the cross-plane direction and the in-plane direction.

### Evaluations of dose algorithm heterogeneity

**Figure 4:**
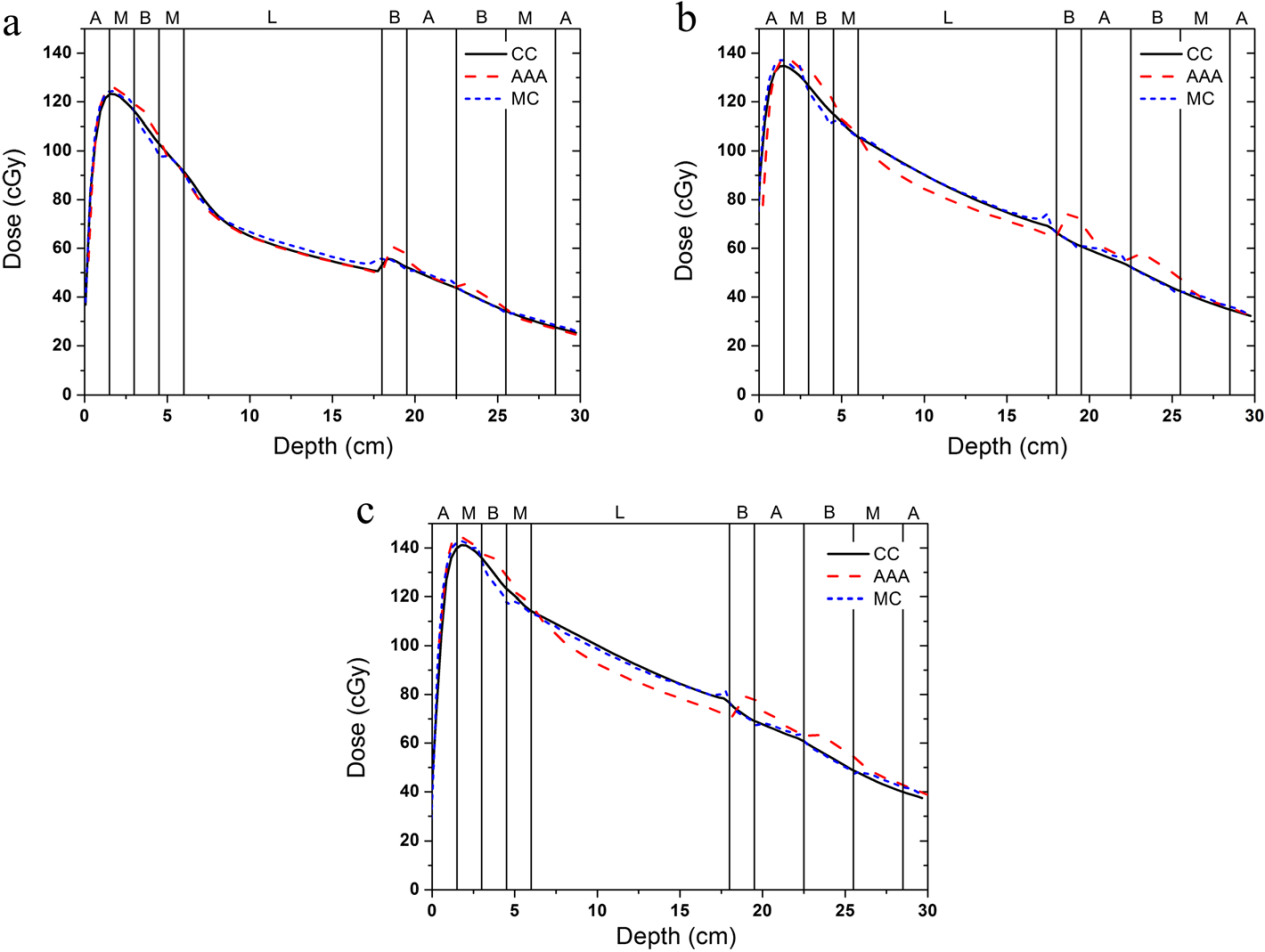
The axial-depth–dose curves in the heterogeneous slab phantom that were obtained using the various algorithms for the 6 MV photon beam and the different field sizes: **a**) field size of 3 cm × 3 cm, **b**) field size of 10 cm × 10 cm and **c**) field size of 20 cm × 20 cm.

**Figure 5:**
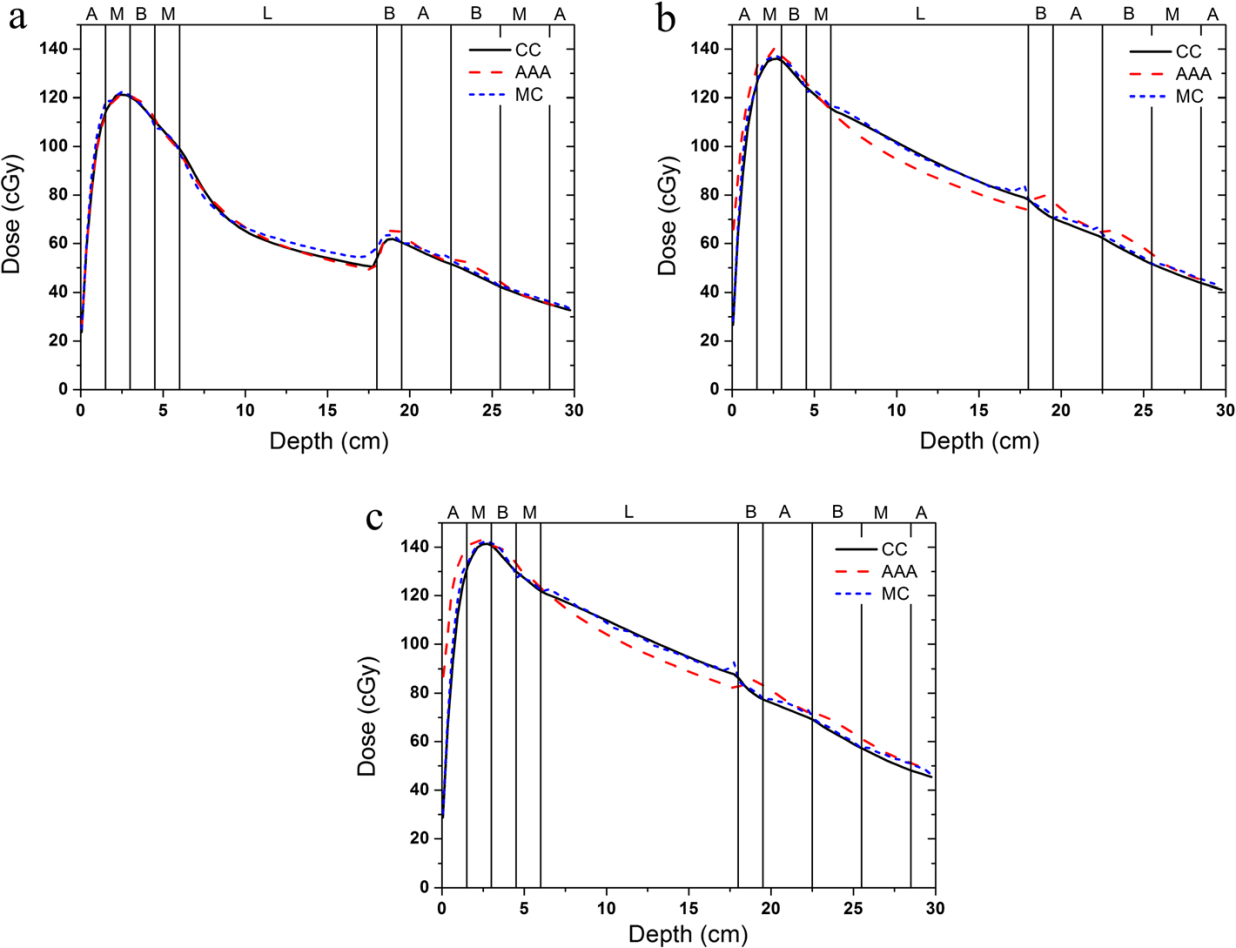
The axial-depth–dose curves in the heterogeneous slab phantom that were obtained using the various algorithms for the 10 MV photon beam and the different field sizes: **a**) field size of 3 cm × 3 cm, **b**) field size of 10 cm × 10 cm and **c**) field size of 20 cm × 20 cm.

Figure 4 presents the axial-depth–dose curves in the heterogeneous slab phantom that were obtained using the various algorithms for the 6 MV photon beam. The percent dose differences presented in this section were normalized to the Monte Carlo simulated doses for a 2 cm depth in the case of the 6 MV beam and for a 5 cm depth in the case of the 10 MV beam; these depths correspond to positions in the muscle slab near the depth at which the maximum dose occurred. The CCCS results agreed well with the results of the Monte Carlo simulation. The dose difference was less than 1.5%, except at the interfaces of different materials. For the AAA, the dose results for the low-density lung region were lower than the Monte Carlo results. The largest dose difference was 7.5%. The dose results for the high-density bone region were higher than the Monte Carlo results, and the largest dose difference was 15%. Figure 5 presents the axial-depth–dose curves in the heterogeneous slab phantom that were obtained using the various algorithms for the 10 MV photon beam. In this case, the CCCS results again agreed well with the results of the Monte Carlo simulation except at the interfaces of different materials. For the AAA, the results were superior to those obtained in the 6-MV case. The largest dose difference in the low-density lung region was 6.4%, and that in the high-density bone region was 9.0%.

### 3D dosimetric verifications

Table 1 presents the gamma pass rates of the gross target volume (GTV), the clinical target volume (CTV), the planning target volume (PTV) and the entire body for the treatment plans. The results for the patient body overridden to water and for the original material are shown. The gamma statistics are within the 10% isodose contours. Good results were obtained using the 3%/3 mm criterion for the planned and EPID-based reconstructed dose distributions in both cases. However, for the tight criterion (2%/2 mm), the pass rates of the original material were decreased compared with the overridden-to-water results; the maximum difference was 22%.

**Table 1:** **The percentage of voxels with a gamma index smaller than 1 for the comparison between the EPID-based results and the TPS results**

**Plan structure**		**Head & neck (6 MV)**		**Lung (6 MV)**		**Lung (10 MV)**		**Prostate (10 MV)**	
		***P***_**w**_ **(%)**	***P***_**m**_ **(%)**	***P***_**w**_ **(%)**	***P***_**m**_ **(%)**	***P***_**w**_ **(%)**	***P***_**m**_ **(%)**	***P***_**w**_ **(%)**	***P***_**m**_ **(%)**
Body	2%/2 mm	74.7	73.2	90.4	83.5	88.9	90.3	94.8	94.1
	3%/3 mm	93.7	93.1	99.8	99.4	99.0	99.1	99.4	99.6
GTV	2%/2 mm	97.5	82.2	94.9	77.4	98.7	76.8	87.8	80.1
	3%/3 mm	100.0	97.5	100.0	96.1	100.0	96.2	97.6	93.0
CTV	2%/2 mm	79.0	71.4	97.3	80.8	99.2	84.8	87.8	83.0
	3%/3 mm	97.8	93.4	100.0	97.2	100.0	97.6	97.6	95.6
PTV	2%/2 mm	73.6	70.1	92.8	79.9	95.5	87.0	92.3	83.9
	3%/3 mm	96.6	92.9	99.7	97.3	99.9	98.5	99.6	98.6

*P*_w_ represents the pass rate of the patient body overridden to water, and *P*_m_ represents the pass rate of the original material.

The detailed statistics of the dose differences for the targets and the OARs of the patient body overridden to water are summarized in Table 2, and the results for the original material are summarized in Table 3. Acceptable agreement was observed between the EPID-based results and the planned results for both the target regions and the OARs. However, the dose differences in the case of the original material were slightly larger than those in the overridden-to-water case. In the case of the patient body overridden to water, the average differences in the mean doses and the maximum doses to the target regions were 0.11 ± 0.52% and 0.32 ± 1.17%, respectively, and the corresponding average values for the OARs were -0.34 ± 1.35% and 0.12 ± 1.74%, respectively. In the case of the original material, the average differences in the mean doses and the maximum point doses to the target regions were 0.57 ± 0.71% and 1.34 ± 0.66%, respectively, and the corresponding average values for the OARs were -0.80 ± 2.05% and 0.41 ± 2.32%, respectively.

**Table 2:** **Comparison between the planned doses and the EPID-based doses for the treatment plans in the case of the patient body overridden to water**

	**Head & neck (6 MV)**		**Lung (6 MV)**		**Lung (10 MV)**		**Prostate (10 MV)**	
	**Diff**_**mean dose**_ **(%)**	**Diff**_**max dose**_ **(%)**	**Diff**_**mean dose**_ **(%)**	**Diff**_**max dose**_ **(%)**	**Diff**_**mean dose**_ **(%)**	**Diff**_**max dose**_ **(%)**	**Diff**_**mean dose**_ **(%)**	**Diff**_**max dose**_ **(%)**
GTV	0.03	1.97	1.16	0.56	0.53	0.56	0.64	0.56
CTV	-0.28	1.98	0.34	-1.49	-0.2	-0.82	0.65	0.66
PTV	-0.52	0.82	-0.27	-1.49	-0.55	-0.82	-0.19	1.41
Brain stem	-0.61	-2.30						
Parotid	2.08	3.09						
Spinal cord	-0.67	-0.72	-1.13	-1.68	-1.89	-1.18		
Both lungs			-0.86	0.03	-1.13	0.14		
Heart			-1.36	-0.52	-1.46	-0.73		
Bladder							0.64	2.48
Rectum							2.61	2.75
Colon							-0.34	0.86

**Table 3:** **Comparison between the planned doses and the EPID-based doses for the treatment plans in the case of the original material**

	**Head & neck (6 MV)**		**Lung (6 MV)**		**Lung (10 MV)**		**Prostate (10 MV)**	
	**Diff**_**mean dose**_ **(%)**	**Diff**_**max dose**_ **(%)**	**Diff**_**mean dose**_ **(%)**	**Diff**_**max dose**_ **(%)**	**Diff**_**mean dose**_ **(%)**	**Diff**_**max dose**_ **(%)**	**Diff**_**mean dose**_ **(%)**	**Diff**_**max dose**_ **(%)**
GTV	0.55	0.64	1.13	1.56	1.15	1.53	1.75	2.48
CTV	0.01	1.08	0.62	0.95	0.65	1.29	1.52	2.20
PTV	-0.38	1.08	-0.50	0.24	-0.24	0.78	0.62	2.20
Brain stem	-4.54	-3.95						
Parotid	1.74	2.95						
Spinal cord	-1.50	-3.15	-2.51	-0.27	-3.07	-0.83		
Both lungs			-0.93	2.03	-1.27	2.18		
Heart			-1.33	-1.13	-1.26	-0.45		
Bladder							1.11	3.45
Rectum							2.94	2.74
Colon							0.95	1.39

### Computation times for dosimetric verifications

Table 4 lists the computation times associated with the various components of the calculation for the GPU implementation, including the initialization of the CUDA subsystem, the fluence-map calculation, the CCCS calculation of one field and the gamma evaluation of the body. According to these results, the total dose computation time for a single field was less than 8 s, and the gamma evaluation was completed in approximately 1 s.

**Table 4:** **GPU computation times for dose calculations and gamma evaluations**

	**Volume (cm** ^**3**^ **)**	**Initialization (s)**	**Fluence map (s)**	**CCCS(s)**		**Gamma evaluation (s)**	
				**TERMA**	**CS**	**Resampling**	**Gamma calculation**
Head & Neck	48 × 30 × 21.6	0.225	0.264	0.030	6.822	0.762	0.821
Lung	54 × 15 × 22.8	0.236	0.263	0.028	5.965	0.522	0.709
Prostate	40.8 × 36 × 31.8	0.231	0.266	0.054	7.324	0.804	0.847

## Discussion

Treatment verification using a 3D dose reconstruction based on information acquired in the treatment room is feasible. EPID-based reconstruction can be used to determine the delivery errors of a treatment plan and to estimate the errors of a TPS dose-calculation algorithm. During treatment, the output of the linear accelerator may differ from the planned delivery determined using the TPS. For example, the positioning accuracy of the multi-leaf collimator may lead to differences in the delivered and planned dose distributions. Because of its high resolution and linear dose response, an EPID can effectively detect the delivery errors of a linear accelerator [36,37]. Accurate and reliable dose-calculation algorithms that consider density inhomogeneities are necessary, especially for the head, neck and thorax [38,39]. For this purpose, the calculation accuracy of the CCCS algorithm for inhomogeneous tissues is acceptable [16,29]. An independent dose-calculation algorithm based on the CCCS algorithm was implemented to successfully estimate the errors of a TPS dose-calculation algorithm. In this study, fluence maps were obtained based on EPID measurements. These fluence maps were used to reconstruct the doses delivered to overridden-to-water phantoms and the original patients. According to the gamma analysis results, good results were obtained for both cases using the 3%/3 mm criterion. The average gamma pass rates were greater than 94%. However, when a tighter criterion (2%/2 mm) was used, the pass rates of the original patients were decreased compared with the pass rates of the overridden-to-water phantoms. The maximum decreases for the head-and-neck, lung and prostate treatment plans were approximately 15%, 20% and 8%, respectively. For both 6 MV treatment plans (head-and-neck and lung), the EPID-based doses to the spinal cord were found to be lower than the planned doses; based on the heterogeneity evaluation, this result can be attributed to the presence of high-density bone material around the spinal cord, which caused the dose results calculated using the AAA to be higher than those calculated using the CCCS algorithm. Fogliata *et al.* have reported similar results [40]. These results indicate that compared with the uniform-phantom-based dosimetric verification method, the complete 3D dosimetric verification method using an independent dose-calculation algorithm can provide more detailed evaluation information.

The advantage of the GPU implementation of the CCCS algorithm is that the 3D dose computation time is significantly reduced, while the necessary accuracy of the dose calculation in inhomogeneous tissues is maintained. These advantages are important for 3D dosimetric techniques that are intended to be applied in labor-intensive clinical verifications. For either pre-treatment or in vivo dosimetric verifications, GPU-based dose reconstruction can achieve true real-time calculations. Chen *et al.* have reported that the single-field computation time can reach 0.5 s when two higher-performance GPUs and a fully optimized CCCS algorithm are used [19]. In this study, for the three different sites considered (which contained 160 × 100 × 72, 180 × 50 × 76 and 136 × 120 × 106 voxels), the single-field dose-reconstruction times were 7 s, 6 s and 8 s, respectively. Thus, our study indicates that there is room for further improvement: 1) High-performance hardware can effectively improve the computation speed. An NVIDIA GTX-765 M notebook video card with 768 CUDA cores was used to perform the computations reported in this study. When we performed the computations on an NVIDIA GTX-TITAN, which contains 2688 CUDA cores, the computation speed was 5-6 times faster than that achieved using the GTX-765 M. 2) Optimization of the CCCS algorithm can also improve the computation speed. Here, we used the lookup method based on tabulated kernels for the calculation. The computation time could be reduced by using the exponential kernel model and the built-in NVIDIA special function unit.

Not only the dose calculation but also the fluence-map reconstruction and the gamma-index calculation were implemented using the GPU. The computation time required to obtain a fluence map from EPID images of 1024 × 768 pixels was reduced to 0.3 s by computing FFTs in parallel on the NVIDIA GPU. By contrast, the computation time that was required when a single CPU was used was approximately 8 s. Wendling *et al.* have presented a fast algorithm for gamma calculation, for which the computation time for a 38.5 cm × 35.3 cm × 25.3 cm dose dataset at a 0.1-cm grid resolution was found to be 28 s [34]. This algorithm still represents a non-negligible time cost for real-time clinical verifications. With the gamma calculation implemented using a GPU, the computation time, including data resampling and gamma calculation, for a 48.0 cm × 30.0 cm × 21.6 cm dose dataset at a 0.1-cm grid resolution was found to be less than 1.8 s.

## Conclusion

A 3D pre-treatment dosimetric verification method based on an EPID was presented. A GPU calculation engine can boost the speed of 3D dose and gamma evaluation calculations, thereby bringing the calculation and evaluation times to the level required for real-time verifications. Although this investigation focused on pre-treatment dosimetric verification, it is our intent to extend the methodology to true real-time 3D dosimetric verification using EPID transit measurements and a GPU calculation engine.

## Consent

Written informed consent was obtained from the patient for the publication of this report and any accompanying images.

## Abbreviations

GPU: Graphic processing unit
EPID: Electronic portal imaging device
IMRT: Intensity-modulated radiation therapy
CCCS: Collapsed-cone convolution/superposition
TERMA: Total energy released per mass
AAA: Analytical anisotropic algorithm
OAR: Organ at risk
DVH: Dose-volume histogram
